# Flexible Composite Films Made of EMAA^−^Na^+^ Ionomer: Evaluation of the Influence of Piezoelectric Particles on the Thermal and Mechanical Properties

**DOI:** 10.3390/polym14132755

**Published:** 2022-07-05

**Authors:** Sandra P. S. Tita, Fernão D. Magalhães, Diana Paiva, Maria A. Z. Bertochi, Guilhermina F. Teixeira, Ana L. Pires, André M. Pereira, José R. Tarpani

**Affiliations:** 1 Materials Engineering Department, Sao Carlos School of Engineering, University of Sao Paulo, Av. João Dagnone, Sao Carlos 13563-120, SP, Brazil; jrpan@sc.usp.br; 2 LEPABE—Laboratory for Process Engineering, Environment, Biotechnology and Energy, Faculty of Engineering, University of Porto, Rua Dr. Roberto Frias, 4200-465 Porto, Portugal; fdmagalh@fe.up.pt (F.D.M.); dianapaiva@fe.up.pt (D.P.); 3 ALiCE—Associate Laboratory in Chemical Engineering, Faculty of Engineering, University of Porto, Rua Dr. Roberto Frias, 4200-465 Porto, Portugal; 4 Department of Biochemistry and Chemical Technology, Chemistry Institute, State University of Sao Paulo, Araraquara 14800-060, SP, Brazil; zaghete@iq.unesp.br; 5 LABEL/FC—Laboratory of Bio-Electrocatalysis and Fuel Cells, Institute of Chemistry, Federal University of Goias (UFG), Goiania 74690-900, GO, Brazil; guilhermina.ferreira@ufg.br; 6 Department of Physics and Astronomy, Faculty of Science, Institute of Physics of Advanced Materials, Nanotechnology and Nanophotonics (IFIMUP), University of Porto, Rua do Campo Alegre, 687, 4169-007 Porto, Portugal; ana.pires@fc.up.pt (A.L.P.); ampereira@fc.up.pt (A.M.P.)

**Keywords:** flexible composite films, ionomers, flexible polymer films-PZT, damage sensor

## Abstract

Studies that aim to produce flexible films of composite materials based on ionomers-PZT, and volume fractions lower than 10% PZT, in order to monitor damage in aeronautical structures are seldom investigated. The growing emphasis on the use of polymers capable of self-healing after damage or activation by heating has motivated the application of self-healing ionomers as polymeric matrices in composites with piezoelectric particles aiming to monitor damage. Flexible composite films were developed based on the self-healing polymer matrix Surlyn^®^ 8940 ionomer (DuPont^TM^—Wilmington, DE, USA) and PZT particles (connectivity 2–3) in volume fractions of 1, 3, 5 and 7%, with thickness around 50–100 µm. The choice of PZT volume fractions followed the preliminary requirement that establishes a final density, which is lower or at least close to the density of the materials used in aeronautical structures. Since the application of composites based on epoxy resin/carbon fibers has been increasing in the aeronautical segment, this material (with density lower than 1500 kg/m^3^) was chosen as a reference for the present work. Thus, due to self-healing (a characteristic of the matrix Surlyn^®^ 8940) combined with recyclability, high flexibility and low thickness, the flexible composite films showed advantages to be applied on aeronautical structures, which present complex geometries and low-density materials. The manufactured films were characterized by SEM, XRD, DMA and mechanical tensile tests. The results were discussed mainly in terms of the volume fraction of PZT. X-ray diffraction patterns showed coexistent rhombohedral and tetragonal phases in the PZT particles-dispersed composite, which can potentialize the alignment of ferroelectric domains during polarization under strong electrical field, enhancing dielectric and piezoelectric properties toward sensing applications. DMA and tensile testing results demonstrated that the addition of PZT particles did not impair either dynamic or quasi-static mechanical performance of the flexible composite films. It was concluded that the PZT volume fraction should be lower than 3% because, for higher values, the molecular mobility of the polymer would suffer significant reductions. These findings, combined with the high flexibility and low density of the ceramic particle-filled thermoplastic polymer, render the developed flexible composite film a very promising candidate for strain and damage sensing in aeronautical structures.

## 1. Introduction

**Figure 1:**
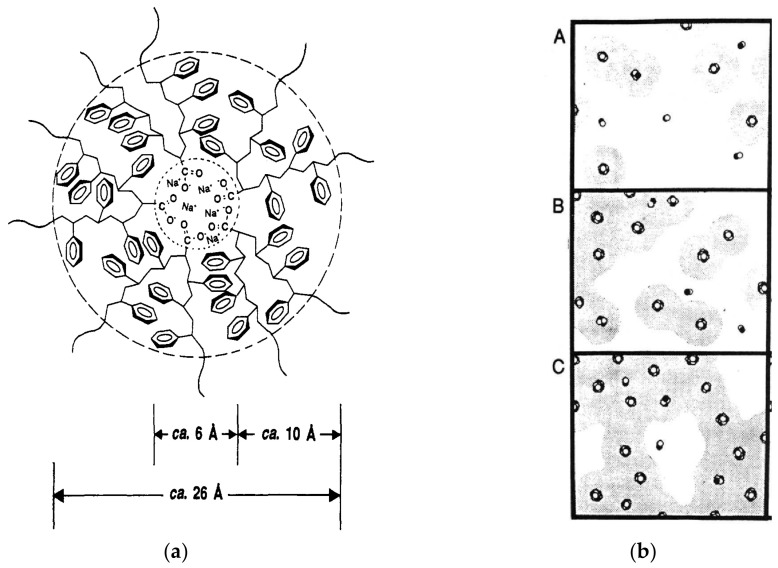
Schematic representation of the (**a**) region of restricted mobility surrounding a multiplet and (**b**) morphologies of random ionomers at different ion contents: (A) low, (B) intermediate and (C) high ion content. Adapted from Ref. [2]. Reprinted with permission from A new multiplet-cluster model for the morphology of random ionomers. A. Eisenberg, B. Hird, and R. B. Moore. Macromolecules 1990 23 (18), 4098–4107. DOI: 10.1021/ma00220a012. Copyright 1990 American Chemical Society.

Ionomers are polymers whose volumetric properties are influenced by interactions between ionic substituent groups covalently linked to the polymeric skeleton, usually in low molar ratio (up to 15% molar), and located in discrete regions of the polymeric material, named as ionic aggregates [1,2]. Some morphological models are found in the literature to elucidate the microstructure of ionomers, and the most accepted being: (a) model of multiplets and clusters [3], according to which ionic species incorporated in the polymeric structure establish interactions in discrete regions, resulting in the formation of ionic aggregates (multiplets) that interact with each other forming clusters; (b) model of order–disorder transitions of ionic clusters [4], in which the clusters suffer first-order transitions during heating at a temperature lower than the melting temperature of the polymer, called temperature of order–disorder; and (c) model of restriction of the mobility of polymeric chains neighboring the multiplets, where it is considered that the chain segments close to the ionic multiplets have lower mobility compared to the chain segments further away from the multiplets, i.e., each multiplet is surrounded by a region of restricted mobility of polymer chain, as if it were a “skin” (Figure 1a). In the last model, known as the EHM model, due to the initial letters of the authors’ names, it was also stated that the greater the ionic content, the greater the number of multiplets distributed in the molecular structure of the ionomer. Therefore, in the EHM model, due to the increase in the density of ions present, the occurrence of overlapping regions of neighboring restricted mobility increases due to the decrease in the distance between the multiplets, which may result in the formation of clusters (Figure 1b) [2]. Morphological models show that ionomers, as a result of their very nature, are supramolecular structures [5] because they have crossover points of physical nature, established by ionic groups (multiplets), which are thermally reversible.

Polymeric ionomers offer a wide spectrum of opportunities for researchers to develop new materials, since the specific behaviors present in these materials (high flexibility, good mechanical properties, excellent adhesion with ceramic materials and regeneration capacity after damage at moderate temperatures), resulting from their molecular structures, enable the investigation of several properties, such as physical, mechanical, molecular dynamics, thermo-dynamic-mechanical, dielectric, thermal and optical, among others [6,7,8,9,10]. Many scientific works explore the concept of piezoelectricity and ferroelectricity using the solid solution of lead titanate and lead zirconate, PbTiO_3_ and PbZrO_3_, respectively. Based on this solid solution, a ceramic system of perovskite structure is obtained, which is also known as lead zirconate titanate [Pb(Zr_0,52_Ti_0,48_)O_3_] or PZT [7,11]. Piezoelectric active PZT exhibits excellent strain reliability and survivability over a large temperature range, from the temperature of liquid nitrogen to the cure temperature of high-performance polymer matrix composite materials, making it worthwhile for in situ health monitoring of aging aircraft fleet throughout the service life [11]. The combination of the properties of polymeric ionomers and piezoelectric ceramic materials, as a perovskite-structured compound, can provide several materials, which are used to the development of smart materials, photovoltaic cells, piezoelectric sensors and biomaterials [7,8,9].

Although PZT has excellent piezoelectric properties, being widely used in transducers, actuators and sensors [12,13], there is a limitation in designing devices exclusively made of this material with complex geometric shapes due to the fragility of ceramic materials. A solution to overcome this limitation, due to the ease of processing (they can be designed in complex geometric shapes), feasibility of reuse (they can be cast and molded several times) and ductility (they can undergo large deformations), is to disperse the ceramic particles in ductile thermoplastic polymeric materials capable of providing attractive mechanical characteristics to different applications (such as increased ductility, improved formability, etc.) without penalizing the sensing capacity of the obtained composite [12]. For instance, PZT has been used in the preparation of composites with the copolymer poly(ethylene-co-methacrylic acid), EMAA, as well as composites employing EMAA-derived ionomers with their acid groups partially neutralized with zinc ions in order to develop sensing materials [12,14]. Given the recognized PZT toxicity, brittleness and relatively high cost [14], blended particulate PZT with thermoplastic polyvinylidene fluoride (PVDF) polymer gives rise to the first piezo film sensor. Regarding the usage of thermoplastic polymers for sensor development, polyvinylidene fluoride, or PVDF, is a widely applied material, as it has superior piezoelectric characteristics/properties compared to other polymers; its piezoelectricity coefficient can reach up to 7 pC/N [15], in addition to being used to obtain efficient triboelectric nanogenerators by improving the polarization of their surface [16]. Moreover, regarding the aircraft applications, it is noteworthy that the materials most used in aeronautical structures are aluminum alloys with density around 2760 kg/m^3^ [17] and composite materials of epoxy resin reinforced with carbon fibers with density approximately 1300 kg/m^3^ [18]. Despite its good piezoelectric properties, the piezoelectric thermoplastic polymer PVDF shows density between 1760 and 1900 kg/m^3^ [19,20], which is lower than aluminum alloys, but it is higher than composites of epoxy resin reinforced with carbon fibers. Thus, it is not considered appropriate that the sensor density is higher than the densities of materials used in aeronautical structures that have large areas, such as panels used in fuselages and wings, which would have to be covered with the piezoelectric composite film. Designing sensors based on piezoelectric composites that combine satisfactory sensing properties with ease of processing and adequate mechanical properties, as well as good flexibility and ability to regenerate damage on a microscale, remains a huge challenge for different fields [14].

Considering the scenario described above, the main motivation of the present work is to monitor structural damage, initially, in aeronautical structures. Thus, flexible films of composites (SNaPZT#) were developed from the regenerable polymer matrix (healing) of Surlyn^®^ 8940 ionomer (DuPont^TM^), based on ethylene/methacrylic acid copolymer, with 5.4 mol% of methacrylic acid groups and 30% of these groups neutralized by sodium ions (Na^+^)—defined as SNa0 in the present work—and PZT particles (connectivity 2–3) in volume fractions (#) of 1, 3, 5 and 7%. The choice of PZT volume fractions followed the preliminary requirement that establishes a final sensor density, which is lower or at least close to the density of the materials used in aeronautical structures. Since the application of composites based on epoxy resin/carbon fibers has been increasing in the aeronautical segment [21], it was chosen as a model in the present work to develop and characterize flexible composite films with density values lower than 1500 kg/m^3^. Thus, it was chosen not to exceed 7% in volume fraction of PZT. In other words, in the present work, the extremely flexible PZT particle-filled ethylene/methacrylic acid copolymer ionomer films are proposed, manufactured and tested to comply with physical, mechanical and thermomechanical requirements for acting as efficient strain and structural monitoring sensors in aircrafts. Therefore, as a first step in this article, it was investigated how the PZT particles can influence the thermal and mechanical properties of the EMAA^−^Na^+^ Ionomer flexible film by using results from scanning electron microscopy (SEM), X-ray diffraction (XRD), dynamic mechanical analysis (DMA) and mechanical tensile tests. In the next paper, not only electrical but also thermal behavior of the flexible composite films will be thoroughly investigated, evaluating their potentialities and limitations to be used as a damage sensor in aeronautical structures.

As scientific contributions, the present work brings relevant data and results related to the new composite films. Thus, it is possible to highlight that the composite possesses very low density, very low particulate volumetric fraction, homogeneous filler distribution, high filler dispersion, high adhesiveness to the substrate and easy forming to contours and corners, in addition to extrinsic self-healing potentialities.

## 2. Materials and Methods

### 2.1. Determination of PZT Mass

Initially, it is very important to perform the preliminary determination of flexible composite films with Surlyn^®^ 8940 as the matrix. Thus, by using the rule of mixture, according to Equation (1), it was possible to determine the volume fractions of PZT in order to obtain flexible films with density lower than 1500 kg/m^3^:

ρ_c_ = ρ_pzt_ϕ_pzt_ + ρ_p_ϕ_p_ (1)

where ρ_c_ is the composite density, ρ_pzt_ is the particle PZT density, ρ_p_ is the matrix Surlyn^®^ density, ϕ_p_ is the volume fraction of Surlyn^®^ matrix, and ϕ_pzt_ is the volume fraction of PZT, considering that ϕ_pzt_ can be equal to 1, 3, 5, 7, 20, 30 or 50%. In the calculus, it was assumed that the density of PZT particles is around 7900 kg/m^3^, and the density of Surlyn^®^ 8940 neutralized by sodium, SNa0, is equal to 950 kg/m^3^.

### 2.2. Production and Characterization of PZT Particles

PZT particles used in the present work were produced using Pechini’s method with molar ratio of Zr/Ti equal to 52/48. They were characterized via scanning electronic microscopy (SEM) with high resolution (JEOL^®^ JSM 7500F, Beijing, China) in order to evaluate the morphology and to determine the individual particle dimensions, as well as the clusters formation. The crystallographic evaluation of PZT particles was conducted by means of X-ray powder diffraction (XRD) using a Rigaku Rotaflex diffractometer model RINT2000 (Beijing, China) with Cu-Kα radiation (λ = 1.5406 Å) with a current of 200 mA and a voltage of 45 kV in the 2θ range of 10° to 80° with an increment of ∆2θ = 0.020° and speed of 5° min^−1^. To perform the analysis, the sample in the powder way was placed inside a cavity with 1 cm diameter in the center of a glass sample holder.

### 2.3. Manufacturing of Flexible Composite Films

#### 2.3.1. Dry Process of the Matrix

The matrix SNa0 was given by DuPont as a pellets shape. Then, before the usage of the pellets, they were submitted to a dry process in an oven with air circulation system under 60–65 °C for 12 h.

#### 2.3.2. Dispersion of PZT Particles via Extrusion Process

For dispersion and distribution of PZT particles in the Surlyn^®^ matrix, an Xplore MC5 (Sittard, The Netherlands) twin-screw micro compounder, with capacity to process material volumes of 5 mL, was used. All composite specimens were processed at 170 °C, under a rotation force of 4.5 kN, with rotation speed equal to 100 rpm (during the feed) and 350 rpm (during the mixture of the materials). The residence time of materials in the extruder during the mixing process was 3–5 min. Based on the calculations shown in Section 2.1, specimens with four different volume fractions of PZT (1, 3, 5 and 7%) were produced, which were named as SNaPZT#, where # corresponds to the value of the PZT volume fraction.

#### 2.3.3. Compression Molding Process: Polymer and Flexible Composite Films

To obtain films of pure polymer (Surlyn^®^ 8940—SNa0) and flexible composites (SNaPZT#), it was used a hot compression molding process of pellets Surlyn^®^ 8940, as well as of extruded composites with different volume fractions of PZT, respectivelly.

**Figure 2:**
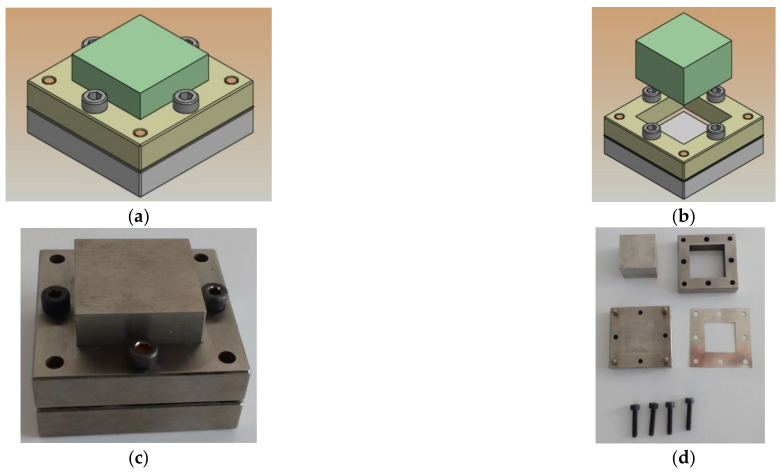

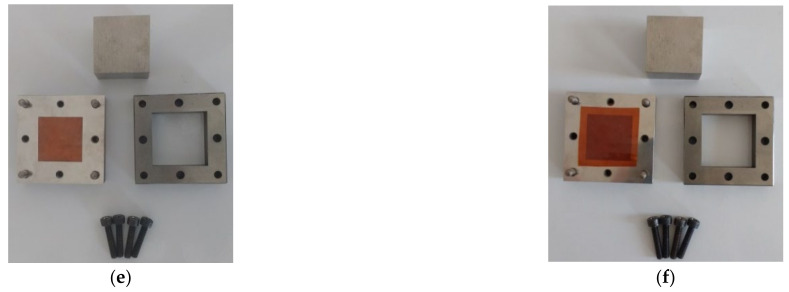
Mold used for the manufacturing process of flexible composite films: (**a**) and (**b**) the design; (**c**) assembled mold; (**d**) mold parts; (**e**) and (**f**) Kapton^®^ films used during the process.

The press system was heated up to 175 °C for 10 min without applying pressure. After that, a pressure of 21.3 MPa per 2 min was applied on a metal mold with specific dimensions (25.4 mm × 25.4 mm × 50 µm—Figure 2) designed and manufactured especially for the present study. During the hot molding process, the Kapton^®^ films (Figure 2a,b) had to be used to aid the release of the films from the molds.

By using the procedure described above, which consisted of three main steps (drying the matrix, mixture of PZT and matrix via the extrusion process and hot compression molding process of the extruded materials), it was possible to produce flexible composite films with specific dimensions and required PZT volume fractions (due to limits of density) in order to have damage sensors able to be applied in aeronautical structures.

### 2.4. Techniques and Methods Used to Characterize the Flexible Composite Films

**Figure 3:**
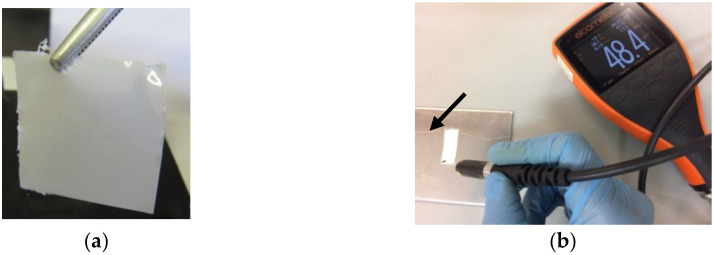
Evaluation of the thickness of the flexible films using the Elcometer^®^ (Manchester, UK): (**a**) flexible composite film (SNaPZT3); (**b**) measurement of film thickness.

The films of pure polymer (Surlyn^®^ 8940—SNa0) and flexible composites with PZT particles (extruded material and films), i.e., SNaPZT#, where # corresponds to 1, 3, 5 and 7% of PZT volume fraction, were investigated by using different techniques and methods, such as SEM, XRD, DMA and mechanical tensile tests. It is important to note that before characterizing the films (Figure 3a), all thicknesses were evaluated using a digital Elcometer^®^ (Manchester, UK), as shown in Figure 3b.

#### 2.4.1. Evaluation of Particle Dispersion via SEM

In order to analyze the quality of particle dispersion, duplicate specimens of the extruded composites, flexible composite films (with 1, 3, 5 and 7% of PZT volume fraction) and pure polymer film were fractured by using liquid nitrogen. The fracture surfaces were covered with gold via the sputtering process. After that, the covered surfaces were analyzed by SEM, using the FEI Inspect^®^ equipment (Hillsboro, OR, USA), models S50 and F-50, which were coupled to an energy-dispersive X-ray spectroscopy (EDS) and to an electron backscatter diffraction (EBSD). All the obtained images were compared with each other.

#### 2.4.2. XRD Analysis Conditions

In order to identify the diffraction peaks related to the crystalline phase, triplicate specimens of the same pure polymer film and duplicate specimens of the same flexible composite films (with 1, 3, 5 and 7% of PZT volume fraction) were evaluated using an XRD Rigaku SmartLab diffractometer at room temperature (RT) over a range of 10–80° in a Bragg–Brentano configuration. Cu Ka radiation with λ = 1.540593 Å, with a current of 200 mA and a voltage of 45 kV, was used. The scan speed used was 3 s per 0.020°. This method was used to identify peaks related to the crystalline phases.

#### 2.4.3. Dynamic Mechanical Analysis Conditions

The storage modulus (E′) and loss modulus (E″), as well as tan δ, given by the relation E″/E′, were determined for the flexible composite films (with 1, 3, 5 and 7% of PZT volume fraction) and pure polymer film.

The dynamic-mechanical behavior with the temperature of the flexible composite films in terms of the PZT volume fractions was investigated. Thus, the influence of the PZT particles was studied in the intervals of temperature related to primary and secondary molecular relaxations due to the movements of the main polymer chains, of the ramifications and/or the lateral groups, as well as other segment movements observed in the matrix Surlyn^®^ 8940 without PZT or SNa0.

For the analyses, the equipment Netzsch^®^—DMA model 242 E Artemis^®^ (Selb, Germany), under traction mode, was used. Duplicate specimens of flexible composite and pure polymer films with rectangular shape 12.00 × 0.05 × 4.00 mm^3^ were evaluated. The tests were carried out from −140 to 60 °C under a cooling rate of 2 °C/min in a controlled atmosphere of nitrogen during both steps of cooling and heating of the specimens.

The loading frequency was equal to 5 Hz with a maximum force of 12 N for maximum amplitude of displacement equal to 40 µm.

#### 2.4.4. Tensile Testing Conditions

The Young modulus (E), the yield stress (σ_y_), the rupture stress (σ_rup_) and the maximum strain (ε_max_) for the pure polymer film and for the flexible composite films (with 1, 3, 5 and 7% of PZT volume fraction) were determined by using mechanical tensile testing. Thus, it was possible to evaluate the influence of the PZT particles on the mechanical response under quasi-static tension loadings.

Specimens of flexible composite and pure polymer films with rectangular shape 25.00 × 0.06 × 10.00 mm^3^ (including end tabs to avoid damage caused by clamps) were evaluated by using the Mecmesin^®^—model MultiTest (Shanghai, China) 1-d equipment, with a load cell of 200 N and speed of displacement equal to 10 mm/min applied monotonically.

During the analyses, engineering stress versus engineering strain curves were considered in order to make the qualitative comparisons of the obtained results for different materials.

## 3. Results and Discussion

### 3.1. Analysis of PZT Particles via X-ray and SEM

**Figure 4:**
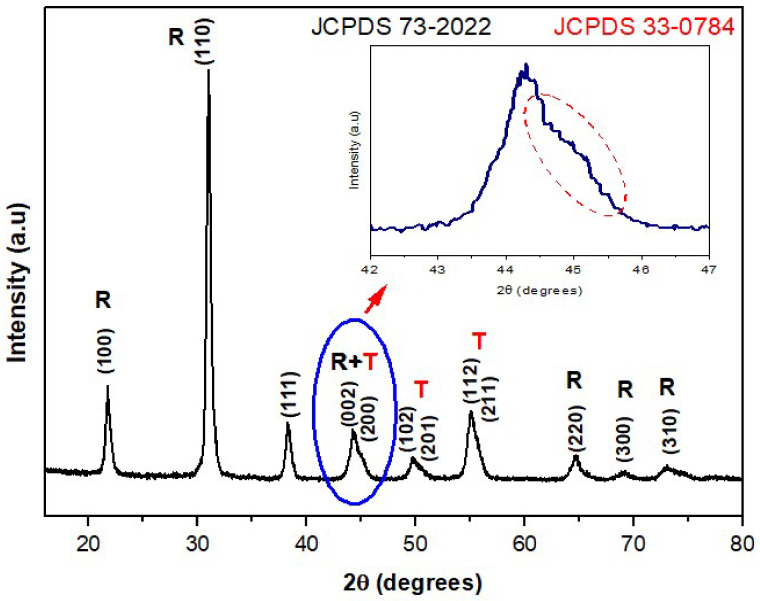
Pattern of diffraction of X-ray for PZT particles obtained via Pechini’s method, highlighting the typical superposition due to the coexistence of tetragonal (T) (JCPDS file n^o^ 33-0784) and rhombohedral (R) (JCPDS file n^o^ 73-2022) phases at the morphotropic phase boundary region.

X-ray diffraction was employed to investigate the crystalline phase of PZT particles. As shown in Figure 4, the XRD pattern of the PZT powder indicates the coexistence of PZT in the tetragonal phase (JCPDS file n^o^ 33-0784) and rhombohedral phase (JCPDS file n^o^ 73-2022). These results corroborate ones reported by James, Mishra et al. and Zak et al. [12,22,23], where a pattern of diffraction of X-ray for the PZT particles, Pb(Zr_0,52_Ti_0,48_)O_3_, was observed, with the coexistence of diffraction peaks related to the rhombohedral phase (200) and to tetragonal phase (002) and (200) in the same region of 2θ between 42° and 47°, as shown in Figure 4, serving as evidence of the morphotropic phase boundary region (MPB) [22,23].

The coexistence of tetragonal phases (T) and rhombohedral phases (R) was confirmed by the X-ray graphic in Figure 4 because, as observed by Singh et al. [24] and by James [12], in the same graphic, there is a presence of a doublet (superposition of two peaks R + T), in the region of 2θ between 44° and 47°, which is related mainly to the tetragonal phase. However, there is also the contribution of the peak in the direction (200) related to the rhombohedral phase. 

Scientific investigations showed that the piezoelectric properties of the PZT particles are improved when there is a coexistence of the tetragonal phase (T) and rhombohedral phase (R) in the morphotropic phase boundary region. This behavior was observed in PZT particles with molar composition of Zr equal to 0.52 [25]. In fact, this is explained due to the presence of a higher number of possible directions for the ferroelectric polarizations present simultaneously when there is a coexistence of phases R and T, there being six directions in phase T and eight directions in phase R [22,26]. It is important to note that the authors assigned the increment in the electromechanical response of PZT to the instability of phase T, in terms of the temperature, in the morphotropic phase boundary region.

**Figure 5:**
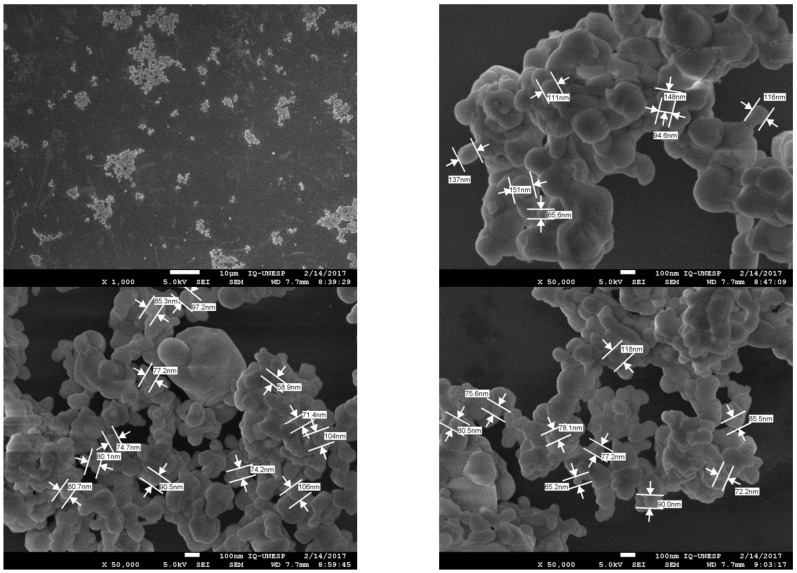
SEM images of the PZT particles obtained via Pechini’s method highlighting the dimensions of some individual particles.

Considering the SEM analyses, it was observed that the PZT particles initially form clusters, although it was possible to take some measurements, as shown in Figure 5, where the values ranged from 0.0652 μm (for individual particles) to 0.262 μm (for clusters). Those measurements were very important in the present work because due to the application of composite films as flexible sensors for damage in aeronautical structures, the films should be around 50 µm thick, considering the PZT volume fractions used. Therefore, during the manufacturing process of flexible films, only PZT particles with maximum dimension of 0.260 μm were used.

### 3.2. Density Values of Flexible Composite Films

To select the PZT volume fractions more suitable for producing flexible composite films for damage sensors in aeronautical structures, first, Equation (1) was used to predict the densities of flexible pure polymer films and flexible composite films represented by $\rho_{sf}^{Na}$.

Second, those predictions of densities were compared to the density of carbon fiber reinforced polymer ($\rho_{CFRP}^{}$) and aluminum ($\rho_{Al}^{}$). Table 1 shows the density ratios between the predicted densities of films and the densities of materials usually used in aeronautical structures, such as composite materials made of carbon fiber and epoxy resin ($\rho_{CFRP}^{}$ ≈ 1300 kg/m^3^) and aluminum alloy ($\rho_{Al}^{}$ ≈ 2760 kg/m^3^).

Observing the results in Table 1, it is possible to conclude that the maximum value of PZT volume fraction for the sensors should not be higher than 7% (SNaPZT7); otherwise, the density of the composite film would be much higher than the composite materials used in aeronautical structures. On the one hand, composite films with 20% of PZT volume fraction could be used as sensors in aeronautical structures made of an aluminum alloy. However, this value of PZT volume fraction must be avoided because, for application in aeronautical composite structures, the density of the sensor would be 1.80 times higher than the density of the structure to be monitored.

### 3.3. Analysis of Distribution and Dispersion of PZT Particles via SEM

**Figure 6:**
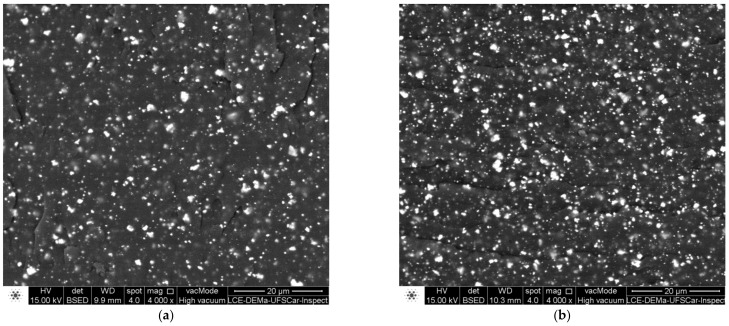

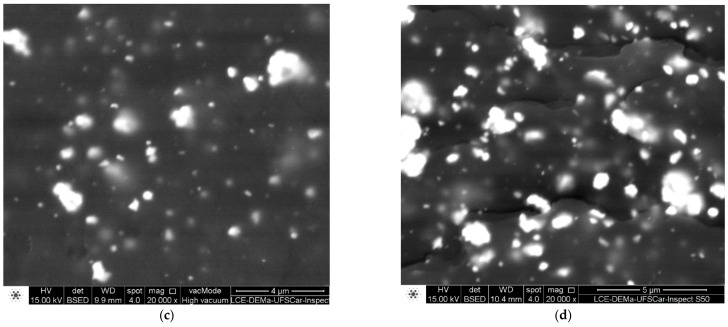
SEM images of fractured surfaces of extruded composites: (**a**) and (**c**) SNaPZT3^e^; (**b**) and (**d**) SNaPZT5^e^ (superscript “e” corresponds to extruded material).

For the extruded composites, the distribution and dispersion of PZT particles in the polymer matrix were analyzed via SEM of the fractured surfaces (Figure 6), which were obtained by fracturing of the material under cryogenic temperature.

The best results for distribution and dispersion were observed for screw rotation speed of the extruder, 300–350 RPM, when the PZT volume fractions were equal to 3 and 5%. In Figure 6, there are SEM images for SNaPZT3 and SNaPZT5 that were obtained using the back-scattered electrons (BSE) mode to eliminate the matrix effect to improve the visualization of distribution and dispersion of PZT particles in the extruded material.

Considering that the signal obtained by BSE is generated via interactions between the primary beam (incident electron beams in the sample), and the electrons present in the regions inside the analyzed material, the images obtained using the BSE mode provide different information. This information depends of the contrast in terms of the topographic image of the region, as well as of the composition in terms of the atomic number of elements in the region [27]. Therefore, the distribution and dispersion of PZT particles in the matrix were better analyzed via SEM by using the BSE mode. Additionally, the images in Figure 6 showed that the distribution and dispersion of PZT particles in the extruded material can be considered adequate, generating a highly homogeneous composite, which will be compressed by using the mold (Figure 2) and a hot press. In other words, this can demonstrate that the methodology for obtaining flexible composite films is successful, showing high potential for obtaining damage sensors with good quality in terms of dispersion of PZT particles.

### 3.4. Characterization of Flexible Composite Films

#### 3.4.1. SEM Results

**Figure 7:**
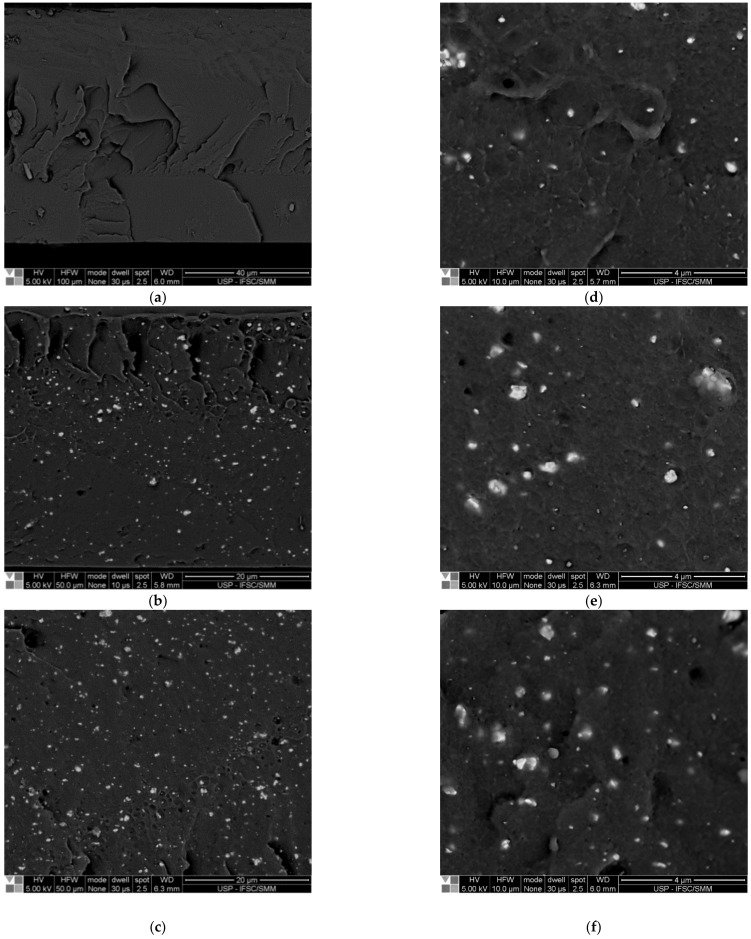
SEM images of fractured surfaces of films: (**a**) SNa0; (**b**,**d**) SNaPZT3; (**c**,**e**) SNaPZT5; and (**f**) SNaPZT7.

The distribution and dispersion of PZT particles in the flexible films were analyzed after hot compression mold processing via SEM of the fractured surface, as shown in Figure 7.

In Figure 7a, there is an image of the pure polymer film SNa0, which can be compared to the images of the flexible composite film with 3% (Figure 7b,d), 5% (Figure 7c,e) and 7% (Figure 7f) of PZT particles volume fraction. The BSE mode was also used during the analysis to verify the distribution and dispersion of particles in the inner regions of the polymer matrix.

Considering that the observed fractured surfaces of the flexible films were along the thickness, the measurements of the thickness of flexible films provided a value equal to 75.0 ± 11.6 µm using a digital Elcometer^®^ (Manchester, UK), as shown in Figure 3b. Additionally, in the SEM images, not only individual PZT particles but also clusters can be verified. For the individual PZT particles, average dimensions lower than 0.10 μm, and for clusters, average values lower than 0.25 μm were observed. Thus, it can be concluded that the dimensions of individual particles and clusters are compatible with the thickness of the flexible films, showing a good distribution and dispersion. Again, this can testify that the methodology for obtaining flexible composite films is successful, showing high potential for obtaining damage sensors with good quality in terms of dispersion of PZT particles.

#### 3.4.2. XRD Results

**Figure 8:**
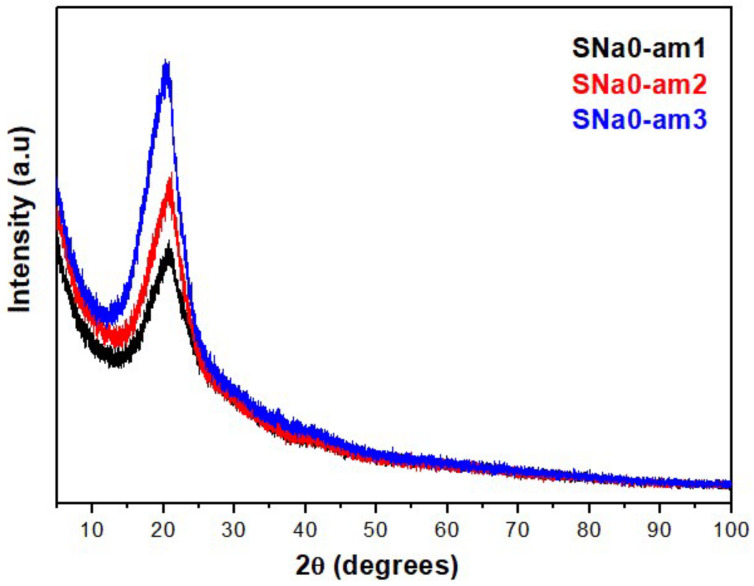
Patterns of diffraction via XRD of three different regions from the same sample of pure polymer film (SNa0).

**Figure 9:**
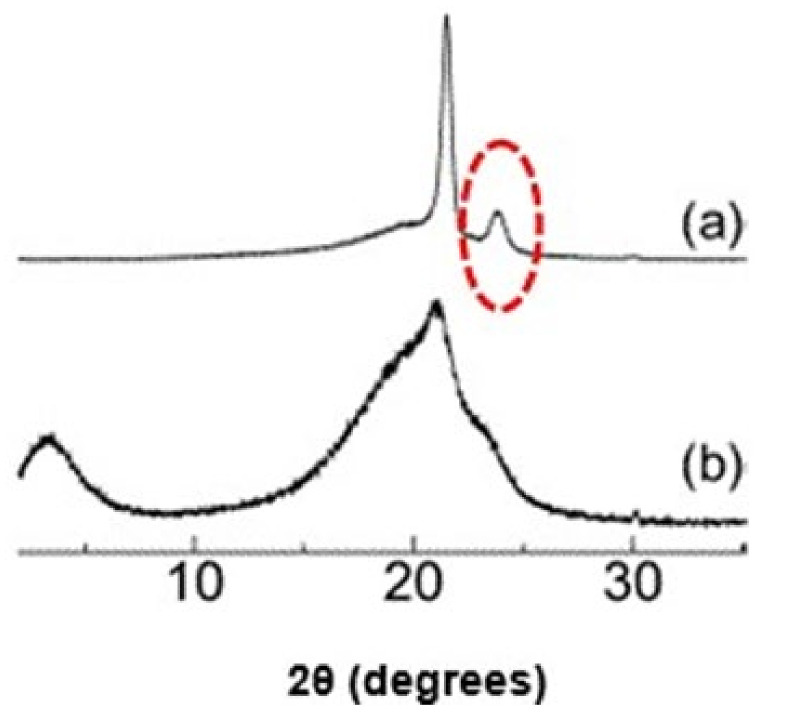
Patterns of X-ray diffraction: (**a**) polyethylene of low density, highlighting the peak of diffraction related to the plane (200) in 23° of orthorhombic crystals; (**b**) copolymer poly(ethylene-co-methacrylic acid) partially neutralized with Na^+^—ionomer EMAA^−^Na^+^. Adapted from Miwa, Kondo and Kutsumizu [29]. Reprinted with permission from Subnanoscopic Mapping of Glass Transition Temperature around Ionic Multiplets in Sodium-Neutralized Poly(ethylene-random-methacrylic acid) Ionomer. Yohei Miwa, Tomoyo Kondo, and Shoichi Kutsumizu. Macromolecules 2013 46 (13), 5232–5237. DOI: 10.1021/ma401035r. Copyright 2013 American Chemical Society.

Figure 8 shows the patterns of diffraction via XRD for pure polymer films (SNa0) and flexible composite films (SNaPZT#). In Figure 8, there are results for three different regions from the same sample of the SNa0 film. In fact, an attempt was made to verify the relevant differences between the patterns of diffraction of a pure polymer film obtained by extrusion and hot compression molding processes. In Figure 8, in the region of 2θ equal to 23°, the absence of one of the typical peaks of orthorhombic crystals of polyethylene regarding the plane (200) was observed. These results are in agreement with the literature [28,29], as shown in Figure 9.

**Figure 10:**
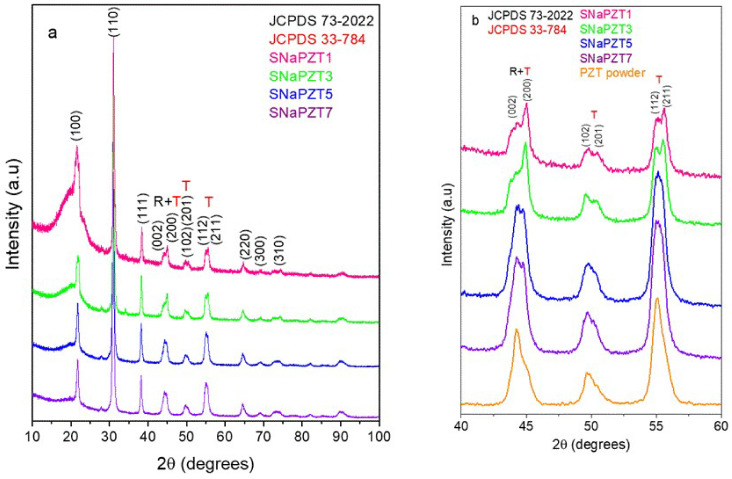
XRD pattern of (**a**) flexible composite films and (**b**) comparison between PZT powder and flexible composite films in the region of 2θ between 40° and 60°.

According to Figure 10, it is possible to note that the presence of the polymeric matrix did not affect the peaks related to the PZT particles (Figure 10a). The XRD results also showed the permanence of peaks of diffraction of PZT particles related to the rhombohedral phase (200) and tetragonal phase (002) and (200) in the same region of 2θ (between 42° and 47°), confirming that they are in the region of morphotropic limit of the phase transition [22,25]. In addition, it was evident that the split peaks regarding the tetragonal phase of PZT (in the region of 2θ between 40° and 60°) were more evident for the composites than the pure PZT particles (Figure 10b).

Previous publications concluded that the ionic interactions present in the multiplets and, consequently, in ionic aggregates, increase the “internal” viscosity of the ionomer, reducing the mobility of the ethylene segments in the chain and avoiding the exclusion of groups related to the methacrylic acid (MAA) and to their derived carboxylate ions (MAA^−^Na^+^) present in lamellae [28,29,30]. This explains the absence of the diffraction peak related to plane (200) at 23°, which is typical for the orthorhombic crystals of polyethylene, as shown in Figure 9a.

Goméz, Gasparini and Canevarolo [31] studied a new morphologic model for explaining the semicrystalline ionomer EMAA^−^Na^+^ by using thermal-optic techniques. They proposed a schema with the presence of groups related to the methacrylic acid (MAA) and their derived carboxylate ions (MAA^−^Na^+^) in the ionomer, before and after thermal aging treatments under dry and humid atmosphere. They observed that the primary crystals of polyethylene were formed by lamellae along their ethylenic segments where there are non-crystallized comonomers of MAA e MAA^−^Na^+^. Those observations can be applied in the present investigation using XRD because the peak of diffraction related to the plane (200) at 23°, which is typical in the orthorhombic crystals of polyethylene, was not observed in the SNa0 film (Figure 8). This occurs due to the presence of non-crystallized comonomers (MAA e MAA^−^Na^+^) in the ethylenic segments that form the lamellae of the ionomer, Surlyn^®^ 8940.

The presence of two phases (rhombohedral and tetragonal) in the samples increases the possibility of alignment of ferroelectric domains during polarization under the application of a strong electrical field (DC—direct current) [22] and, consequently, better dielectric and piezoelectric properties [32].

#### 3.4.3. DMA Results

By using DMA, it was possible to analyze the thermomechanical properties of the flexible pure polymer film (SNa0) and flexible composite films (SNaPZT#), as well as the influence of the PZT particles volume fraction on the thermomechanical responses.

The dimensions of the DMA specimens are described in Section 2.4.3 (Dynamic Mechanical Analysis Conditions).

According to Canevarolo Jr. [33], there are different methods for evaluating the temperature of the same transition, such as: (1) the temperature at the beginning of the fall down or at the extrapolated beginning (T_*E*′*onset*_) of the storage modulus curve (E′); (2) the temperature at the beginning or at the peak (T_*E*″*peak*_) of the loss modulus curve (E″); (3) the temperature at the beginning or at the peak of the damping curve (tan δ); (4) the extrapolated temperature of the change at baseline (in the format of “S”) in the differential scanning calorimetry (DSC) curve. Table 2 shows the results for glass transition temperatures of SNa0 and SNaPZT# films via DMA, which were determined with two different methods (1 and 2). In fact, the glass transitions occur due to the different morphological transformations, devitrification of the regions rich in ions and fusion of secondary crystals, which take place simultaneously, promoting gradients of glass transition temperatures in different regions of the ionic clusters, according to the thorough investigation carried out by Miwa, Kondo and Kutsumizu [29].

**Figure 11:**
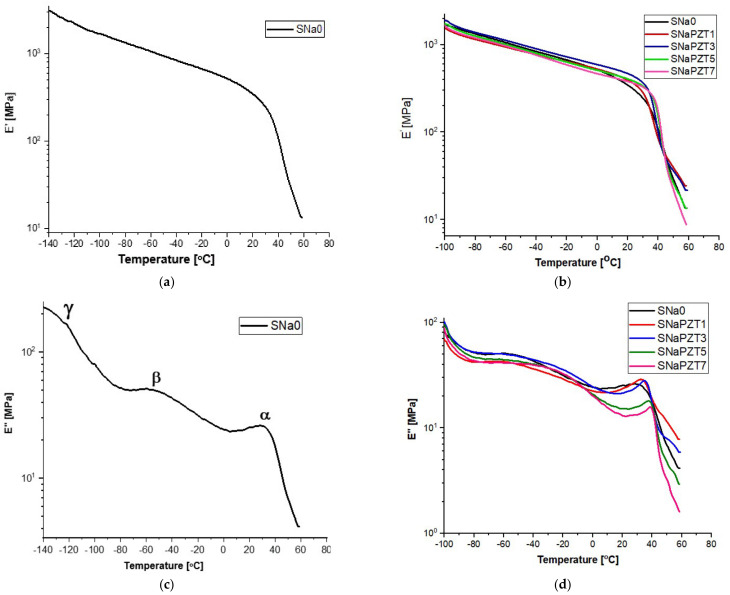
DMA curves: (**a**) E′ for SNa0 film; (**b**) E′ for different SNaPZT# films; (**c**) E″ for SNa0 film (with α, β and γ relaxations); (**d**) E″ for different SNaPZT# films.

In Figure 11, it is possible to observe the DMA results for the SNa0 and SNaPZT# films. The storage modulus (E′) of SNa0 (from −140 °C) and SNaPZT# (−100 °C) from the films is shown in Figure 11a,b, respectively. The loss modulus (E″) of SNa0 (from −140 °C) is shown in Figure 11c. Different curves of the loss modulus (E″) of SNaPZT# (from −100 °C) are shown in Figure 11d, highlighting the α, β and γ relaxations.

Comparing the curves in Figure 11, it was observed that the addition of PZT particles produced only small changes in the viscoelastic relaxations of films. According to the literature [34,35,36,37,38], for ionomers of EMAA neutralized by ions Na^+^, the γ relaxation is related to the molecular movements of short segments of the polymer chain in the amorphous phase. α and β relaxations are related to the neutralization by ions Na^+^ and indicate that there is a separation of phases between the ionic aggregated and the polymer phase of the hydrocarbon matrix, with the consequent formation of ionic clusters. α relaxation is frequently assigned to the glass transition due to the microbrownian molecular movements of the main chain segments close to the region of ionic clusters. Longworth and Vaughan [28] already reported a drop in the storage modulus (E′) and a peak in the loss modulus (E″) in the temperature range of α relaxation in the ionomer that was only dry annealed at room temperature. Those changes were related to the dependence between the relaxation of the ethylene chain segments and the ionic interactions, being significantly present in these temperature ranges. Wakabayashi and Register [35,39] interpreted the α relaxation as a consequence of the melting of secondary crystals and the creation of breaks (points of failure) in the rigid pathway, causing the acceleration of reduction in the storage modulus (E′).

β relaxation is related to the movement of long segments of the amorphous region of the branched polyethylene phase and to the rotation around the linkages of lateral groups to the main chain. Therefore, in the present investigation, α and β relaxations were associated with the individual molecular movements that occur in the ionic clusters (α relaxation) and in the polyethylene chain (β relaxation). Deschanel et al. [36] also mentioned that β relaxation can be related to the response of glass transition of the copolymer non-neutralized in the amorphous phase, which was considered as belonging to the restricted mobility region.

The peaks in the curves of the loss modulus (E″) (Figure 11c,d), between 30 and 45 °C (α relaxation), are related to the molecular relaxations due to the transition order–disorder of the ionic content or due to the glass transition in the long segments of the polymer chains in the regions of ionic clusters, as shown by Kalista Jr., Pflugc and Varley [40] and Miwa, Kondo and Kutsumizu [29]. With the increase in the volume fraction of PZT, there was an increase in the temperature at which α relaxation (α transition peak) occurs and the magnitude of E″ increases (Figure 11d), which is an indication of the reduction in molecular mobility with the addition of PZT.

**Figure 12:**
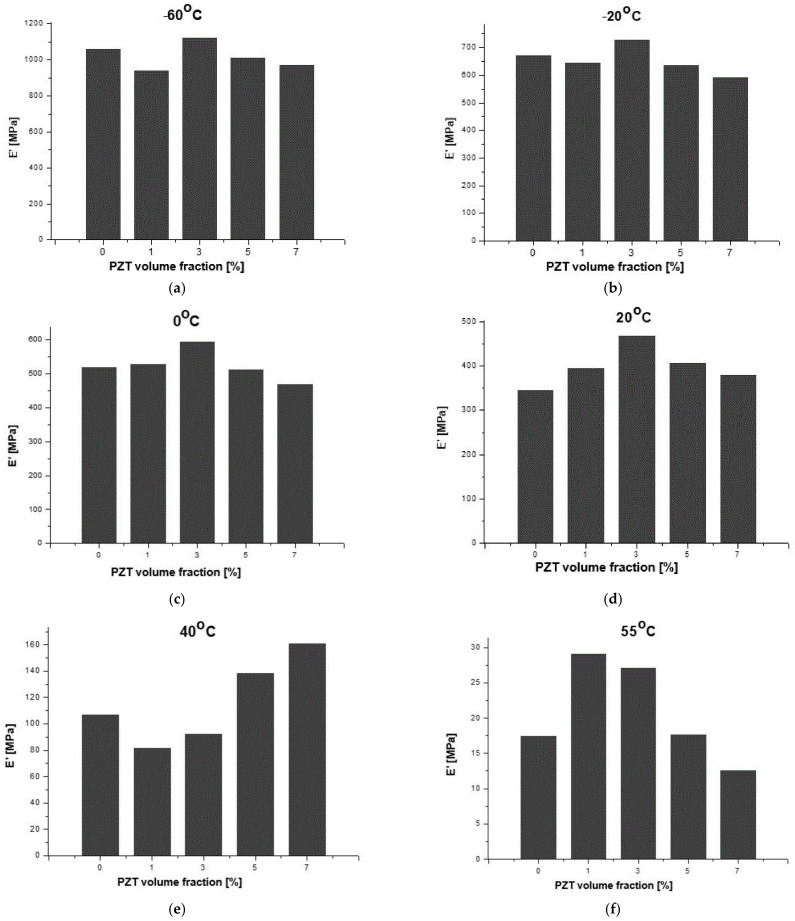
Qualitative evaluation of storage modulus (E′) of SNa0 and SNaPZT# films at different values of temperature: (**a**) −60 °C, (**b**) −20 °C, (**c**) 0 °C, (**d**) 20 °C, (**e**) 40 °C and (**f**) 55 °C.

Considering the results for the SNa0 film as a reference in the present investigations, in Table 3, it is possible to observe the percentage variations of storage modulus (E′) in terms of temperature for different PZT volume fractions when compared to the respective results for the SNa0 film at the same temperature. It should be noted that the results shown in Table 3 and Figure 12 are only qualitative and only indicate trends. It is well known, mainly in the aeronautical field, that it is necessary to continue the work with quantitative analysis of the results, using statistical methodologies of the data obtained from tests of a greater number of replicates.

Regarding the aeronautical applications for the sensors and observing Figure 12, from −60 °C to 20 °C, which consists of a critical interval of temperature for the evaluation of applications of the SNaPZT# composite flexible films for monitoring damage at low temperatures in an aircraft in service, it was verified that SNaPZT3 showed a tendency to increase this property. This suggests that there is better interaction between the matrix and the particles when the PZT volume fraction is equal to 3%. At a temperature of 40 °C (Table 3 and Figure 12e), within the range of temperatures in which an α relaxation occurs, the composite films show a tendency of opposite responses in terms of percentage variation of the storage modulus (ΔE′) when compared to the results under other temperatures shown in Table 3 and in Figure 12a–d,f (i.e., under temperatures of −60, −20, 0, 20 and 55 °C). A greater reduction in the storage modulus (E′) was observed for the composites SNaPZT1 and SNaPZT3, around 24.0% and 13.0%, respectively, which indicates a smaller interference of these PZT volume fractions (1 and 3%) in the relaxation of ethylene chain segments and, therefore, in the microbrownian molecular movements of the main chain segments close to the region of ionic clusters. Even so, SNaPZT3 remains very attractive for monitoring damage in aircraft at low temperatures (between −60 and 30 °C). The same previous considerations are highlighted regarding the need to carry out quantitative investigations. Therefore statistical analysis of the dispersion of values obtained after testing of a greater number of replicates can be perfomed.

Investigations via solid-state Nuclear Magnetic Resonance (NMR), using the ^13^C cross-polarization magic angle spinning (^13^C CPMAS) technique, were carried out by Costa et al. [41] and showed that, for flexible composite films with 3% of PZT volume fraction, the signal related to the segments of -CH_2_- in the amorphous regions of the polymer chain practically did not vary. However, for flexible composite films with 7% of PZT volume fraction, the intensification of the signal in the amorphous segments was verified, which is coherent with the DMA results. This occurs because more intensive perturbations were observed in the segments of the polymer chain, as shown in Figure 11d. At a temperature of 20 °C, the SNaPZT3 film showed the highest percentage variation of the storage modulus E′ (in Table 3) compared to the other films (35.5%).

The same authors also used the ^13^C dipolar chemical shift correlation (^13^C DIPSHIFT) technique, at 83 °C [41]. Those studies tried to identify the molecular origin of dynamic transitions for the flexible composite films. It was verified that the amorphous portion of the polymer chain for the SNaPZT7 films showed the highest reduction in molecular mobility due to the higher PZT volume fraction. Moreover, Costa et al. [41] concluded that the PZT volume fraction in the flexible composite film should be lower than 3% because, for higher values, the molecular dynamic of the polymer would suffer significant reductions.

**Figure 13:**
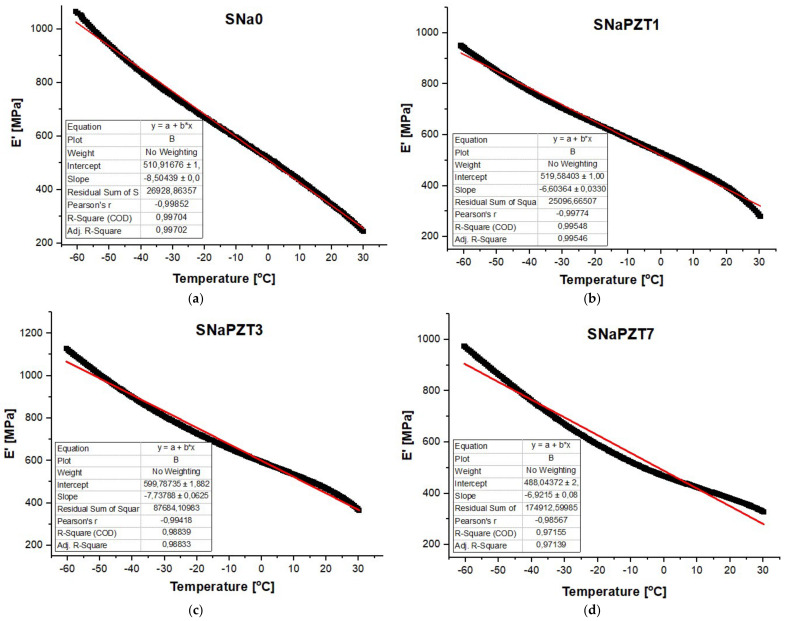
Linear behavior of storage modulus (E′) in terms of temperature: (**a**) SNa0, (**b**) SNaPZT1, (**c**) SNaPZT3 and (**d**) SNaPZT7.

Figure 13 shows that there is a linear behavior of the storage modulus (E′) in terms of temperature for the SNa0 and SNaPZT# films. The interpolation results for 0, 1, 3 and 7% of PZT volume fractions were observed. The result for 5% is not available, but it is similar to the others. This kind of behavior is very interesting for the development of theoretical models, which aid in predicting the thermo-mechanical behavior of the sensors when applied in the aeronautical structures, considering the investigated range of temperature. This is another contribution for the real application of the sensors in the aeronautical field, where the structure can be from −50 °C to 30 °C.

### 3.5. Tensile Testing Results

The dimensions of the films for tensile testing did not follow any specification given by technical standards due to the limitations on manufacturing the flexible films in the present study. However, during all tests, it was guaranteed that the sample was only under uniaxial tensile loading. Moreover, end tabs were used to avoid the failure of the samples close to the clamps. For each type of film (SNa0 and SNaPZT#), five rectangular samples with 25.00 mm length, 0.06 mm thichness and 10.00 mm width were tested.

**Figure 14:**
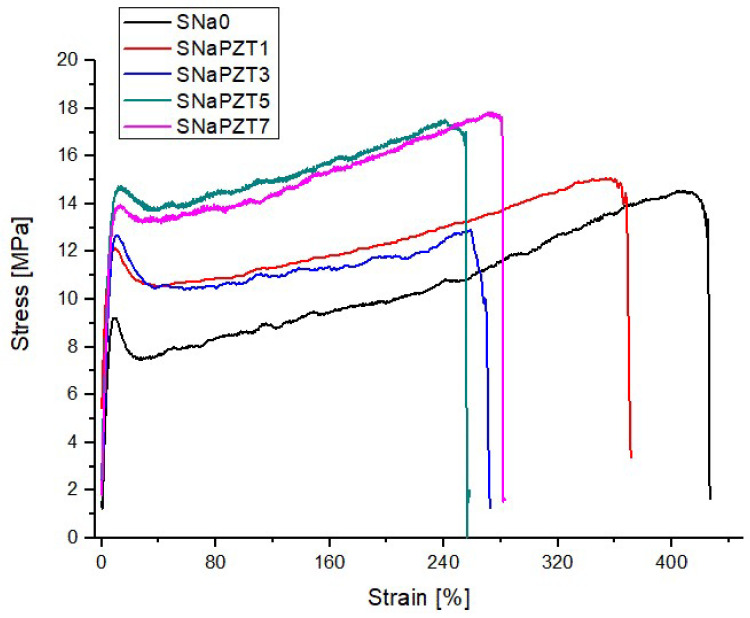
Tensile stress–strain engineering curves for SNa0 and SNaPZT# films.

After the tests, the engineering stress and strain were calculated to perform a comparison of the different films in terms of the PZT particle volume fractions. Figure 14 shows the typical tensile stress–strain engineering curves obtained for the SNa0 and SNaPZT# films. In Table 4, it is possible to observe the mechanical properties of the films obtained from the stress–strain curves. As expected [14], there was a relevant reduction in the maximum strain values with the increment of the PZT volume fractions. Despite of the addition of PZT particles, the films were still ductile, which is a very important characteristic for damage sensors.

As expected, the addition of PZT particles reduced the molecular mobility as discussed previously in the DMA results and in the investigations of Costa et al. [41]. This is reflected in the increment of the Young modulus (E) for all SNaPZT# films (Table 4).

The rupture stress value provided by DuPont for SNa0 (pure polymer), 33 MPa, is higher than twice the average value obtained in the present work, 15.31 MPa. It is important to note that the characterization of the material carried out by DuPont followed the dimensions of samples specified by technical standards, with much larger values.

Regarding the yield stress results, Deschanel et al. [36] and Scogna and Register [37] showed information and discussion about the yield stress for the copolymer EMAA. Those authors pointed out that the value of yield stress can be related to a combination of different mechanisms, such as: (a) material strength due to the yield of rigid domains (crystalline regions); (b) material strength due to intermolecular interactions in the amorphous phase (this is very important if T < T_g_ or T≈T_g_, and if the test speed is high); (c) material strength due to the strain and orientation of the molecular net in the amorphous phase (net of chains linked or not to crystalline and amorphous domains). The investigations by Eisenberg et al. [2] about ionomers similar to EMAA had confirmed that there is a high difficulty in relaxation of the amorphous polymer segments in the vicinities (around) of ionic aggregates. On the other hand, under very low strain rate, the yield stress values are higher. In other words, lower values of yield stress can be related to the plasticity response of the polyethylene crystals and the incomplete relaxation of the amorphous segments of the polymer chains in the regions of restricted mobility. In Table 4, it is verified that the yield stress increases with the increment of PZT volume fractions, although significant increments are not observed. Therefore, the obtained yield stress values indicate that the presence of PZT particles created more difficulties for the relaxation of the amorphous segments of polyethylene chains in the regions of restricted mobility, as confirmed by NMR results published by Costa et al. [41] and by using the results of the DMA.

### 3.6. Comparative Data with Traditional Materials

Composites made of ferroelectric polymers (e.g., PVDF) with ceramics particles (e.g., PZT) are widely studied for application in hydrophones, generation of images in the biomedical area with ultrasound and applications in non-destructive testing [42].

Regarding PVDF, in accordance with the theories of polymer physics, there are no studies in the literature, which confirm the presence of necessary and sufficient secondary interactions to establish a supramolecular structure. Consequently, PVDF cannot be considered as a material capable of self-healing/healing damage at the early stages.

Some properties and characteristics of the Surlyn^®^-type thermoplastic matrices (such as the ionomer EMAANa or SNa0) and the PVDF matrix were compared in Table 5. The density of the Surlyn^®^ 8940 matrix (EMAANa type), equal to 950 kg/m^3^, which was determined by the manufacturer DuPont Packaging & Industrial Polymers [43], is lower than the PVDF matrix, as highlighted in [19], which is equal to 1900 kg/m^3^, and in [44], it provides values between 1740 and 2000 kg/m^3^. Dynamic-mechanical thermal analysis (DMTA) studies showed wide temperature intervals referring to the different relaxations present in the ionomers (β and α relaxations), for example, for the SNa0 matrix, between −60 and 50 °C [35]. Considering the transition temperature gradients referring to the segments of polymeric chains present inside and outside the regions of restricted mobility of the SNa0 ionomer matrices [29], different possibilities of application of these materials can be explored. Although the EMAANa-type matrix has a lower melting temperature, when compared to the melting temperature of PVDF, the self-healing capacity gives an additional advantage for the use of the former (EMAANa) in terms of recyclability. In addition, the Young modulus of SNa0 is more than four times higher when compared to PVDF (Table 5), and it is almost six times higher for flexible composite films.

Therefore, there are some differences between PVDF and the Surlyn^®^/PZT ionomer composites (flexible composite films) studied in this work, such as: (a) in PVDF, there is no self-healing with initial damage; (b) the densities of PVDF are much higher than the Surlyn^®^/PZT composites. In fact, the density values of PVDF exceed those found in composites for aeronautical applications, for example, those based on carbon fiber/epoxy resin, which have densities around 1300 kg/m^3^.

Considering the aspects pointed above, it is shown that the flexible composite films present high potential to be applied in composite structures.

## 4. Conclusions

Aiming to monitor structural damage in aeronautical structures, flexible films of composites were developed from the self-healing polymer matrix (healing) based on ethylene/methacrylic acid copolymer—Surlyn^®^ 8940 ionomer (DuPont^TM^)—and PZT particles (connectivity 2–3) in volume fractions of 1, 3, 5 and 7%. As the first step, it was investigated how the PZT particles can influence thermal and mechanical properties of the composite films by using the results from scanning electron microscopy (SEM), X-ray diffraction (XRD), dynamic mechanical analysis (DMA) and mechanical tensile tests.

The composite materials were produced by melt mixing. After that, the composite films were obtained by hot compression molding. Considering the discussion of the results, the composite flexible films with 3 and 5% of PZT particles volume fraction showed the best results in terms of particle dispersion within the polymer matrix. Moreover, the DMA and tensile testing results demonstrated that the addition of PZT particles did not impair the thermomechanical and mechanical properties of the flexible composite films (SNaPZT#). Therefore, the obtained results (e.g., density, Young modulus and range of temperature for applications) are very promising for the development of damage sensors for aeronautical structures (made of CFRP and with large dimensions), mainly when compared to traditional materials, as shown in Table 5.

## Figures and Tables

**Table 1:** Density of flexible pure polymer film SNa0 and flexible composite films SNaPZT#, where # is equal to 1, 3, 5, 7, 20, 30 and 50% of PZT, represented by $\rho_{sf}^{Na}$ .

Flexible Films	Volume Fraction SNa—ϕ_p_^Na^ [%]	Volume Fraction PZT—ϕ_pzt_ [%]	Film Density—$\rho_{sf}^{Na}$ [kg/m^3^]	Density Ratios *	
				$\rho_{sf}^{Na}$/$\rho_{CFRP}^{}$	$\rho_{sf}^{Na}$/$\rho_{Al}^{}$
SNa0	100	0	950.0	0.73	0.34
SNaPZT1	99	1	1019.5	0.78	0.37
SNaPZT3	97	3	1158.5	0.89	0.42
SNaPZT5	95	5	1297.5	1.00	0.47
SNaPZT7	93	7	1436.5	1.11	0.52
SNaPZT20	80	20	2340.0	1.80	0.85
SNaPZT30	70	30	3035.0	2.33	1.10
SNaPZT50	50	50	4425.0	3.40	1.60

(*) Density ratios between predicted densities of ($\rho_{sf}^{Na}$) pure polymer film (SNa0) or flexible composite films (SNaPZT#) and composite material ($\rho_{sf}^{Na}$/$\rho_{CFRP}^{}$) or aluminum alloy ($\rho_{sf}^{Na}$/$\rho_{Al}^{}$).

**Table 2:** Comparison between the results for T_g,_*_clusters_* (or t*_ord-disord_*) of SNa0 and SNaPZT# films via DMA by using two different methods.

Material	T_*E*′*onset*_ (°C)	T_*E*″*peak*_ (°C)
SNa0	28.8	30.8
SNaPZT1	29.3	32.9
SNaPZT3	32.8	35.2
SNaPZT5	36.3	37.8
SNaPZT7	37.3	38.9

**Table 3:** Percentage variations of storage modulus (E′) in terms of temperature (obtained via DMA) for SNaPZT# films, considering values of SNa0 film as reference.

Material	ΔE′ (%)					
	−60 °C	−20 °C	0 °C	20 °C	40 °C	55 °C
SNaPZT1	−11.1	−3.8	1.4	14.3	−23.8	67.4
SNaPZT3	6.0	8.7	14.3	35.5	−13.3	55.7
SNaPZT5	−4.5	−5.3	−1.5	17.8	29.7	1.3
SNaPZT7	−8.5	−11.9	−9.9	10.2	50.9	−27.6

**Table 4:** Mechanical properties of SNa0 and SNaPZT# films obtained from the tensile stress–strain engineering curves.

Material	E [MPa]	ΔE [%]	^1^σ_y_ [MPa]	Δσy [%]	^2,3^σ_rup_ [MPa]	Δσ_rup_ [%]	^3,4^ε_max_ [%]	Δε_max_ [%]
SNa0	136.1 0 ± 23.93	0.00	10.05 ± 1.28	0.00	15.31 ± 2.88	0.00	404.10 ± 95.50	0.00
SNaPZT1	175.69 ± 22.33	29.09	12.53 ± 1.10	24.68	14.38 ± 2.93	−6.07	245.23 ± 145.06	−39.31
SNaPZT3	162.09 ± 21.31	19.10	12.38 ± 1.81	23.18	13.21 ± 0.69	−13.72	269.00 ± 107.58	−33.43
SNaPZT5	156. 55 ± 15.33	15.03	13.54 ± 1.16	34.73	15.58 ± 2.92	1.76	266.67 ± 23.36	−34.01
SNaPZT7	169.15 ± 14.11	24.28	13.73 ± 1.43	36.62	16.01 ± 1.36	4.57	235.87 ± 29.19	−41.63

^1^σ_y_ = yield stress; ^2^σ_rup_ = rupture stress; ^3^reference values from DuPont: σ_rup_ = 33 MPa and ε_max_ = 470%, where ^4^ε_max_ = maximum strain.

**Table 5:** Comparison of properties and characteristics: ionomer EMAA_Na_ versus PVDF.

Properties	EMAA_Na_ or SNa0	PVDF
Density [kg/m^3^]	950 ^a^	~1900 ^b^ 1740 (amorphous phase) and 2000 (crystalline phase) ^c^
Temperatures [°C]	−60 and 50 (α and β relaxations) ^d^	−35 (glass transition) ^d^
T_melting_ [°C]	94 ^a^	160–165 ^d^
Young modulus [MPa]	136.1	29.5 ^e^
Self-healing	yes	no

References: ^a^ [43], ^b^ [19], ^c^ [44], ^d^ [35] and ^e^ [45].
